# What does learning together mean for pharmacy and medicine students: is it really about from and with?

**DOI:** 10.15694/mep.2018.0000110.1

**Published:** 2018-05-28

**Authors:** Josephine Thomas, Koshila Kumar, Anna Chur-Hansen

**Affiliations:** 1 University of Adelaide; 2 Flinders University

**Keywords:** Interprofessional, Health professional education, Medical Education, Pharmacy

## Abstract

This article was migrated. The article was marked as recommended.

Healthcare students from different professional backgrounds are often brought together under the banner of Interprofessional Education (IPE) in an effort to improve collaborative practice. Despite the demonstrated positive impact of IPE on students’ knowledge, skills and attitudes, it is not clear what students think about learning with students from another health profession. The aim of this study was to explore pharmacy and medicine students’ views and experiences of learning together.

Participants were Year 3 Pharmacy and Year 4 Medicine students, with qualitative data gathered via a written reflection.

Three main themes were identified. Students were accepting of learning with the other professional group. Learning about was evident, particularly in relation to each other’s roles and contributions to patient care. Learning from another professional group was the most problematic as students tended to view and treat knowledge as a commodity to be acquired from another rather than something that could be jointly developed.

While medicine and pharmacy students’ valued learning with and about each other, they were less likely to engage in co-constructing and sharing new meanings and thus learn from one another. To provide a basis for meaningful collaborative practice, IPE needs to challenge students’ fundamental assumptions, beliefs and values about learning with, from and about other professions.

## Introduction

Greater collaboration between pharmacy and medicine is linked to demonstrated improved patient outcomes, particularly in the management of chronic disease ( Daniels 2008, Gallagher and Gallagher 2012). This is a particular necessity in the pharmacotherapeutics context, as the increase in available medications and multi-morbid patients add to the complexity of patient management. Polypharmacy is a common situation --and the likelihood of drug interactions for these patients, increases with multiple medications ( Barton et al. 2012, Roughead et al. 2013). The resulting therapeutic regimens are difficult for a single practitioner to navigate safely and require a multifaceted and collaborative approach.

A collaborative approach to care involving multiple healthcare professionals is a complex undertaking for several reasons, including: power relationships; need for common language; professional culture; workflow and workload pressures. Although medicine and pharmacy share similar roots and many common values, the two professions have evolved separate cultures and different scopes of practice ( Austin et al. 2007, Gallagher and Gallagher 2012, Gilbert 2001). The traditional relationship between them is unequal and a power gradient is evident, with medicine as the dominant profession, afforded by societal perceptions of physicians as saving and prolonging lives ( Austin, Gregory and Martin 2007, Barrow et al. 2011). Despite the potential to contribute to patient safety, the pharmacist’s role is seen as subordinate to the physician’s role ( Routledge 2012). In keeping with this power gradient, most pharmacists are reluctant to question a physician’s authority and opinion about prescribing even though they have a more detailed knowledge of drug properties, interactions and effects, by virtue of their training ( Rosenthal et al. 2010). This entrenched hierarchical relationship between pharmacy and medicine makes it difficult to establish practice that is truly collaborative. In addition, changes in the nature of pharmacy practice over recent years, may further exacerbate the conflict between the professions due a perceived need to protect their own professional territory ( Rosenthal, Austin and Tsuyuki 2010).

Interprofessional education (IPE) is an approach to enhancing the contact and learning between different professional groups in order to improve the future collaborative practice of health professionals ( Greene et al. 1996). The widely accepted definition of IPE is where “.. students from two or more professions learn about, from and with each other to improve collaboration ..” ( Health Professions Network Nursing and Midwifery Office 2010). Many studies have demonstrated positive impacts of IPE on health professional students’ attitudes, knowledge, skills and behaviours; and in some cases these have been shown to translate into later practice ( Reeves et al. 2016, Reeves et al. 2008, Tolleson et al. 2016). Furthermore, the literature shows that students’ attitudes to IP practice often improve after contact with another professional group ( Van Winkle et al. 2012, Whitehead and Kuper 2012). Students also rate IPE as a positive experience; with the overarching sentiment that they believe that IPE is worthwhile. Despite an abundance of evidence regarding the outcomes of IPE, what is missing is a more nuanced understanding of what pre-registration students think and experience in learning with students from another professional group. This study explores pharmacy and medicine students’ views and experience of learning with another health profession. We posed the research question: what and how do students think they learn with, from and about each other?

## Methods

The relevant institutional Ethics Committee granted ethics approval.

### Context and participants

The participants in this study were undergraduate pharmacy and medical students from two universities in Australia. The medical student cohort comprised 198 year 4 students and the pharmacy cohort comprised 114 year 3 students. Undergraduate pharmacy and medicine programs in Australia have predominantly secondary school leaver entry and are 6 and 4-year programs respectively. Students provided written consent and participation in the research was voluntary.

Forty-three students participated in the study. The mean age of medical students was 21 years (range 20-24), 58% female; pharmacy students mean age = 22 years (range 19-32), 76% female.

### Data collection

Data were collected via a reflective writing activity. This activity was designed to probe participants for their views about learning with students from another healthcare profession.

### Data analysis

All reflective pieces were de-identified and assigned a unique ID number by administrative staff. Data were analyzed using a thematic analysis approach, as outlined by Braun and Clarke ( Braun and Clarke 2012). Analysis involved a number of interrelated steps including: familiarization with the data, reading and rereading. Inductive coding of individual pieces was then performed. The second and third authors reviewed the first author’s analysis, sampling the raw data, to determine congruence between reported themes and ensure no themes were missed. Themes, subthemes and codes were listed in a matrix with illustrative quotes from individual participants for each code. Later, codes were collapsed where it was apparent that there were similar themes or clustering of themes. Coding was performed until saturation was reached, which was after a total of 38 reflective pieces (19 medical and 19 pharmacy). The codes were grouped into subthemes and themes linked explicitly to the research question.

### Researcher reflexivity

The insider position of the first author, as a clinician from a General Internal Medicine and Clinical Pharmacology background working in an interprofessional team environment, and as a university academic responsible for designing and implementing IPE, afforded first-hand knowledge of the setting and the participants which was invaluable in interpreting the study findings. This intimate knowledge related to the curriculum; the culture within the medical program and clinical practice environments; and the relationship between teachers and practitioners in pharmacy and medicine. The other authors had little familiarity with participants and the setting, and this enabled a balance of insider and outsider perspectives to inform the interpretations made in this study.

Ethics approval was granted by relevant Ethics Committees of The University of Adelaide (15/02), The University of South Australia (03/15) and Flinders University (OH-000-47).

## Results

Three main themes were identified related to what medical and pharmacy students’ view and experiences are in learning with students from another professional group. These were: “Learning with” which incorporates the emotional language used to describe the contact between groups, the levels of comfort and familiarity with the other group as well as linkages drawn to contact between professions in other settings. “Learning from” which includes students’ recognition of complementary skillsets and field of knowledge of the two professional groups. “Learning about” which encompasses the expression of views about their own and other professions’ role in the healthcare team, the notion of a professional hierarchy and the power differences between them. Illustrative quotes are presented for each theme (Participant ID: M= medicine, P= pharmacy).

### Learning with

Students reported they were generally comfortable in learning with other profession and welcomed the opportunity to learn with a different professional group. They tended to frame the contact between professional groups in positive emotional language, including the adjectives: interesting, enjoyable, enlightening, happy and valuable. One source of transient apprehension and discomfort for some students was the unfamiliarity of students from the other professional group. Some pharmacy students reported that contact with the other group made them more comfortable in challenging the traditional power relationship between them, but it is unclear if they would enact this in practice. Some students reported that learning with another professional group had enhanced their appreciation of how to communicate with the other professional group. Both medicine and pharmacy students could see the value in learning together with another profession before graduation, because of the need to work together later. Many students drew links between better patient outcomes and the team approach to clinical practice.

“They were very nice people who had similar [sic] chosen a similar path to us medical students and so had similar priorities and values”(M15).

“I will be more proactive and less intimidated by the status of a doctor [physician]” (P36).

“hope both professions could work more closely together than they currently are because i think it will result in better medical care” (P31).

### Learning from

Both pharmacy and medical students recognized the complementary nature of the knowledge base of the two groups’. However, an interesting contradiction emerged as the students spoke about their level of knowledge and contributions. Pharmacy students tended to see their own knowledge deficits as barriers to engagement and collaboration with another profession, while medical students viewed their knowledge deficits as an area for improvement rather than an impediment to collaboration. Students described plans to increase their own knowledge by studying resources such as past lectures, books, online tools and modules. Medical students in particular flagged an intention to utilize ward pharmacists as a resource in the clinical setting to bridge gaps in knowledge for patient care, but it was not clear how they would go about this activity. “Any time that there is a pharmacist attached to the team I am on, I will ask lots of questions about drugs that I don’t understand, and medication regimes for different diseases. ...to broaden my knowledge” (M3).

Another contradiction also emerged in how medical and pharmacy students thought about each other’s knowledge. While some medical students perceived pharmacy students as highly knowledgeable, with greater knowledge and depth of understanding on specific areas, particularly basic pharmacology, others made judgments about pharmacy students’ relative lack of clinical experience, inferior levels of knowledge and inability to apply knowledge in clinical settings, which they felt prevented interaction as equals. “I felt that the pharmacy students were lacking in knowledge in key aspects that prevented them from making equal contributions compared to myself and my medical student partner. Even when I outright prompted the pharmacy students for their thoughts, too often they struggled to make a substantial comment” (M10).

In contrast, pharmacy students were less likely to talk about medical student knowledge deficits, but some did note that medicine students’ relative lack of detailed medication knowledge was not befitting the prescribing role of a physician. “it makes you realise how little doctors [physicians] know about medicines and their specifics. It’s not their fault as its not really in their curriculum, but its scary when you consider they’re allowed to prescribe and we’re not” (P31).

### Learning about

The physician as leader was a common theme. Medical students saw themselves as leading the engagement between professional groups and parallels were drawn with the professional hierarchies observed in their clinical experience. There was a perceived need to prompt and push the other professional group reflecting a sense of arrogance and superiority. Medical students articulated the physician’s role within the healthcare team as that of coordinator, gatekeeper and final arbiter, determining which other professionals should be involved and how.

The role of pharmacist was clearly articulated as a medication expert, but there was a clear sense this was a subordinate role to that of the physician, reflecting in the use of words such as; “support”, “assist”, “aid”, “advise”, “suggest”. Students perceived the pharmacists’ main role was to act as a safety net for physicians as in terms of providing a second check in the prescribing process. This safety net role was most clearly articulated by pharmacy students. Students expressed how a pharmacist could add to patient care through their role in implementing a physician’s plan, mostly by advising patients on optimal use of medicines. “As a pharmacist, realistically, we are to double check that what the doctor [physician] has prescribed and avoid potential errors. Unrealistically, we would take part in the prescribing decision to help decide the best pharmacological treatment, if needed, for the patient.”(P26).

The concept of the pharmacist (and allied health professionals more generally) providing a different and complementary perspective on the patient’s care was expressed, although this was not always seen as a positive attribute and some medical students were dismissive of the different approach. “they have a very different perspective on patient care. In addition, the ‘pharmacist’ seemed to want to limit the number of medications to minimize side effects rather than add medications to treat all the conditions which was interesting. This seemed to demonstrate a theoretical understanding rather than adapting to a real-life situation where multiple disease processes and prioritization is required” (M9). Integration of pharmacy students’ input into therapeutic regimes and medication choices was seen to be at the discretion of the physician, i.e. able to be dismissed or ignored. “You should ask for the pharmacist/other allied health where appropriate of their specific options and try and endorse that where possible. However, you have to make the final decision on what is most appropriate for the patient” (M14).

## Discussion

Undergraduate pharmacy and medical students were largely positive about learning with and about another profession. Students could see benefits to patients and benefits to their future practice in learning about their professional roles and those of their colleagues. However, students did not appear to value and invested little effort in co-constructing understandings and creating shared knowledge. Medical students demonstrated a marked propensity toward assuming the role of leader and saw this as part of both their scope as learner and group participant, as well as part of their professional role. Pharmacy students overwhelmingly adopted a subordinate role, providing information support and viewed their professional role as advisory, providing verification, rather than active co-contributor.

The traditional relationship of physician as the dominant professional appeared in reflections of both groups of students. The concept of the pharmacist making important contributions to patient care by fulfilling a safety check role, was recognized by both groups; but perhaps more emphatically by the pharmacy students. Nevertheless there was an undertone that questioning a physician’s authority is difficult for a pharmacist. There was some questioning of the power imbalance, particularly in relation to prescribing; where the superiority of physicians was seen as inappropriate when medicine students’ detailed knowledge is seen as inadequate for the task. This is seen as a mismatch of capabilities and responsibilities, since pharmacists do not have the right to prescribe; yet their knowledge of medications is better than the medical practitioners. Although understandable this attitude is somewhat incongruent with the poor uptake of increased responsibility that has been available to pharmacists in recent years, including limited prescribing rights ( Chan et al. 2008, Roberts et al. 2005, Rosenthal, Austin and Tsuyuki 2010). Nevertheless, the prescribing role is a major component of the power gradient between the professions, with the medical prescriber perceived as having the greater responsibility and the pharmacist role as supporting the prescriber.

Knowledge appears highly valued by these undergraduate students and used as a measure of professional worth. This is evident in the medical student reflections, which praised the pharmacy students as “medication experts”; but can also be appreciated, in the derogatory comments about pharmacy students’ lack of knowledge. Pharmacy students themselves also cited inadequate knowledge as a reason for their lack of confidence to contribute meaningfully to learning with, about and from other professional groups. When medical students mentioned learning from another professional group, this was described in terms akin to a “taking of knowledge”. They expressed the intention to utilize (as distinct from work collaboratively with) pharmacists to bridge gaps in their knowledge. Overall, this study lends support to the notion that knowledge is seen and treated as a commodity by undergraduate healthcare students as something to be taken or utilized, rather than something to be jointly developed.

Overall, this study illustrates that there are a number of issues associated with undergraduate students learning “with, from and about” each other. These seem to reflect traditional power differences and professional hierarchies between the professions, and can impede meaningful interprofessional learning. Whilst contact between professional groups can provide a platform for deeper learning, this is more likely to happen if students experience challenges their assumptions about other professions and their beliefs about the value around interprofessional interaction ( Mezirow 1997). Learning from others can only occur if participants are open and willing to new perspectives ( Hovey and Craig 2011). It requires the learners to co-construct and share new meanings, which does not occur in this study. Some authors have suggested that IP practice requires greater development of the self and may therefore be a longer-term proposition beyond licensure ( Ward et al. 2017).

This lack of “learning from” does not fit with how we as educators, tend to conceptualize IPE. However, from a practical perspective it illustrates the complexity of ensuring the desired outcomes when we put students from different professions together ( Kuper and Whitehead 2012). Learning with others, has enabled both groups to learn something about the other profession; and they perceive this as worthwhile. Perhaps two out of three is sufficient, since learning with and (a little) about is enough to enable professionals to work together. The literature may be wrong about how we conceptualize IPE. D’Amour and colleagues have stated that in order to collaborate one must be familiar with the other professions’ roles, responsibilities and conceptual models.( D’Amour and Oandasan 2005) Whilst the end goal of collaborative practice is certainly valid, achieving the requisite familiarity with another professional group could be seen as a necessary first stage ( Charles et al. 2010).

This study provides in depth insight into how undergraduate healthcare students perceive learning with students from another health profession. However, there are limitations to this study, including that it relies on self-reported data from students within one academic institution in Australia obtained at only one time point and social desirability response bias ( Fisher 2000). While the insider position of the first author intimately shaped the research approach and interpretations, the co-authors who were outsiders provided a useful counterbalance in interpreting the findings.

## Conclusion

Learning from another professional group requires greater openness to co-construct and share new meanings and was not achieved in this IPE setting. Learning with another professional group is seen as positive by learners; and enables an understanding of roles and responsibilities in patient care. Some learning about another profession occurs in IPE and this small shift in attitudes will likely have benefits for future practice. It may provide the foundations for building collaborative practice at a later stage. However, it is unlikely this will be sufficient on its own to result in the significant advancement of a more collaborative model of practice, in the context of wider influences and set patterns of professional roles and relationships. To provide learners with the understandings that can form the basis for collaborative practice, we need to challenge their fundamental assumptions, beliefs and values around other professions and interprofessional interaction.

## Take Home Messages

• Students in the health professions value learning *with* and *about* each other.
• In order to learn *from* one another, students need to be willing to engage in co-constructing and sharing new meanings.
• Professional hierarchies and power differentials can impede meaningful interprofessional learning

## Notes On Contributors

• Josephine Thomas is a practicing General Internal Medicine Specialist and Clinical Pharmacologist. She is a Clinical Educator and PhD candidate in the School of Psychology and School of Medicine, Faculty of Health and Medical Sciences, University of Adelaide, South Australia 5005
• Koshila Kumar is a Senior Lecturer in the Prideaux Centre for Research in Health Professions Education, at Flinders University, South Australia 5042. She is an experienced qualitative researcher and has published on a range of topics in health professions education.
• Anna Chur-Hansen is Head of School of Psychology, Faculty of Health and Medical Sciences, University of Adelaide, South Australia 5005. She is a Registered Psychologist who has taught across a range of health professions since the 1980s.
