# Supplementary material for: What does learning together mean for pharmacy and medicine students: is it really about from and with?
Source: MedEdPublish (2016). 2018 May 28;7:110. [Version 1] doi: 10.15694/mep.2018.0000110.1 (PMC10699384; doi:10.15694/mep.2018.0000110.1)
Supplement: Peer review history

# Reviewer report: Reviewer response for version 1

**DOI:** 10.21956/mep.19582.r29065

**Published:** 2018-06-14

**Authors:** Megan Anakin

**Affiliations:** 1 University of Otago

This review has been migrated. The reviewer awarded 4 stars out of 5 I found this clearly written article informative and thought-provoking. Understanding interprofessional education from the student perspective will allow educators and researchers to enhance their efforts to design and implement interprofessional learning activities that will help students to develop an appreciation for and skills to engage in collaborative practice once they are working in healthcare. As a previous reviewer noted, to enhance the usefulness and implications of the findings for practice, the authors could make explicit links between their findings and the CAIPE https://www.caipe.org/ definition.There are a few minor inconsistencies to possibly address in the manuscript. For example, in the methods section, ethical approval could be addressed once instead of twice. In the first sentence there is a general statement ethical approval and in the last sentence specific details of ethical approval are given. Is the following statement correct? “Undergraduate pharmacy and medicine programs in Australia have predominantly secondary school leaver entry and are 6 and 4-year programs respectively.” In New Zealand, our programmes in medicine are longer than in pharmacy.Thank you for conducting this study. It will add to our understanding of IPE.

Reviewer Expertise:

NA

No decision status is available

# Reviewer report: Reviewer response for version 1

**DOI:** 10.21956/mep.19582.r29064

**Published:** 2018-05-30

**Authors:** Shelley Fielden

**Affiliations:** 1 Leeds Institute of Medical Education

This review has been migrated. The reviewer awarded 4 stars out of 5 This is a well written and interesting paper which frames an IPE intervention in the context of the CAIPE definition of IPE (learning with, from and about) to explore the extent to which student participants evaluated the learning opportunity. The paper highlights the role of entrenched professional hierarchies and the extent to which changing roles and responsibilities may challenge and/or reinforce these and the extent to which IPE can help manage this change. It would have been helpful to have had some further context on what types of IPE the students involved in the study had participated in to give the reader some background as to whether students’ views were based on formal teaching, informal learning on placement and the frequency of their experiences of IPE etc. I would encourage the authors to look at the literature from the UK and other countries where the role of the prescriber has been extended beyond the sole responsibility of the doctor given the recent changes to prescribing in Australia in considering how to develop this project further.

Reviewer Expertise:

NA

No decision status is available

# Reviewer report: Reviewer response for version 1

**DOI:** 10.21956/mep.19582.r29063

**Published:** 2018-05-28

**Authors:** P Ravi Shankar

**Affiliations:** 1 American International Medical University

This review has been migrated. The reviewer awarded 4 stars out of 5 This is an interesting and well-written paper. Interprofessional education (IPE) is becoming more common all over the world. The authors examine IPE from the perspective of the learners. The methods section is well described. The authors divided participant’s experience about IPE into learning with, learning about and learning from. The medical and pharmacy students had problems with learning from each other and in co-constructing knowledge. The nature of the relationship between pharmacy and medical students described in the paper is commonly seen in developing nations. Though Australia has a long history of pharmacist involvement in patient care, the role of the pharmacist is still mainly seen as a safety net for the doctor. Pharmacy students were of the opinion that medical students may not have adequate knowledge about medicines. The authors also mention that pharmacists are slowly gaining prescribing rights for a limited number of medicines. This manuscript will be interesting reading for health professions educators.

Reviewer Expertise:

NA

No decision status is available
